# Mobile device for thrombolysis decisions for telestroke

**DOI:** 10.25100/cm.v49i4.3921

**Published:** 2018-12-30

**Authors:** Antonio J Salazar, Nicolás Useche, Manuel F Granja, Aníbal J Morillo, Sonia Bermúdez, Didier Sossa, Claudia J Ortiz, Oscar J Torres, Brenda Ropero

**Affiliations:** 1 Universidad de los Andes, Laboratorio de Telemedicina y Electrofisiología. Bogotá, Colombia; 2 Fundación Santa Fe de Bogotá, Hospital Universitario . Bogotá, Colombia; 3 Baptist Neurological Institute, Lyerly Neurosurgery, Jacksonville. FL, USA.; 4 Universidad El Bosque, Facultad de Medicina. Bogotá, Colombia.

**Keywords:** Infarction, mobil device, middle cerebral artery, stroke, cerebrovascular disorders, neurologists, intracranial hemorrhages, tomography, X-ray computed, aneurysm, computers, handheld, arteriovenous, malformations, Infarto, Tele radiologia, dispositivo movil, arteria cerebral media, accidente cerebro vascular, enfermedad cerebrovascular, neurologo, hemorragia intracraneal, tomografia, rayos-x computarizado, aneurisma, computadores, equipo portátil, arteriovenoso, malformaciones

## Abstract

**Aim:**

This study compares the reliability of brain CT interpretations performed using a diagnostic workstation and a mobile tablet computer in a telestroke context.

**Methods:**

A factorial design with 1,452 interpretations was used. Reliability was evaluated using the Fleiss’ kappa coefficient on the agreements of the interpretation results on the lesion classification, presence of imaging contraindications to the intravenous recombinant tissue-type plasminogen activator (t-PA) administration, and on the Alberta Stroke Program Early CT Score (ASPECTS).

**Results:**

The intra-observer agreements were as follows: good agreement on the overall lesion classification (κ= 0.63, *p*<0.001), very good agreement on hemorrhagic lesions (κ= 0.89, *p*<0.001), and moderate agreements on both without acute lesion classification and acute ischemic lesion classification (κ= 0.59 and κ= 0.58 respectively, *p*<0.001). There was good intra-observer agreement on the dichotomized-ASPECTS (κ= 0.65, *p*<0.001).

**Conclusions:**

The results of our study allow us to conclude that the reliability of the mobile solution for interpreting brain CT images of patients with acute stroke was assured, which would allow efficient and low-cost telestroke services.

## Resumen

**Objetivo:**

Este estudio compara la confiabilidad de interpretaciones de TAC de cráneo simple realizadas utilizando una estación de trabajo de diagnóstico y un computador tableta en un contexto de teleACV.

**Métodos:**

Se utilizó un diseño factorial con 1,452 interpretaciones. La confiabilidad se evaluó utilizando el coeficiente kappa de Fleiss en las concordancias sobre los resultados de la interpretación en la clasificación de la lesión, la presencia de contraindicaciones en la imagen para la administración intravenosa del activador del plasminógeno tisular (AP-t) y con el *Alberta Stroke Programme Early CT Score* (ASPECTS).

**Resultados:**

Se obtuvieron las siguientes concordancias intraobservadores: buena concordancia en la clasificación general de la lesión (κ= 0.63, *p*<0.001), muy buena concordancia en lesiones hemorrágicas (κ= 0.89, *p*< 0.001), y concordancia moderada en ambos sin clasificación de lesión aguda y clasificación de lesión isquémica aguda (κ= 0.59 y κ= 0.58 respectivamente, *p*<0.001). Hubo una buena concordancia intraobservadores en el ASPECTS dicotomizado (κ= 0.65, *p*<0.001).

**Conclusiones:**

Los resultados de nuestro estudio permiten concluir que la confiabilidad de la solución móvil para la interpretación de imágenes de TAC de cráneo simple de pacientes con accidente cerebrovascular agudo (ACV) estaba garantizada, lo que permitiría servicios de teleACV eficientes y de bajo costo.

## Introduction

Patients who arrives at emergency services with acute stroke symptoms, are diagnosed and treated using a well establish and standardized protocol, named *the stroke code*, for which several tasks are undertaken. One of the main outcomes for this group of patients is determine whether to treat the patient with tissue plasminogen activator (t-PA) administration or whether to perform an endovascular thrombectomy. As part of this protocol, a non-contrast brain computed tomography (CT) examination is performed ^1^ ^,^ ^2^ and its interpretation is achieved by experienced neuroradiologists, who in a first step must establish if there are no contraindications for the t-PA administration based on the provided images. Further, in addition to the neurologist’s expertise, the patient´s treatment is determined, which is crucial for the patient’s outcome.

In the brain CT interpretation the patient’s lesion is be classified as one of the following: hemorrhagic lesion, acute stroke, chronic stroke, or without acute lesion. A hemorrhagic lesion is a contraindication to the t-PA administration; other contraindications arrive if there are signs such as the presence of intraaxial neoplasm, intracranial neoplasm, arteriovenous malformation, aneurysm, hemorrhagic transformation of an ischemic infarct. Besides, if the patient´s Alberta Stroke Program Early CT Score (ASPECTS) ^3^, which is used for assessing the size of the infarct on brain CT scans , is less than or equal to six, the patient is not eligible to the t-PA administration ^1^ ^,^ ^2^ ^,^ ^4^ ^,^ ^5^ as there is a high risk of malignant middle cerebral artery infarction after the t-PA administration ^6^. For these reasons, interpretations of brain CT of patients with acute stroke sings in our hospital are performed by neuroradiologists with significant expertise in stroke. However, in most hospitals in our country, there are no neuroradiologists, at all. Similarly, our location, which is a Joint Commission International (JCI)-certified primary stroke center with endovascular thrombectomy capabilities, there are no enough of them to support a telestroke network. In order to increase the supply and availability of neuroradiologists, mobile solutions using tablet computers may be developed.

The aim of this study was to evaluate the reliability of brain CT scan readings performed by means of a primary diagnostic interpretation workstation and a mobile tablet computer in the context of a Colombian emergency telestroke network. According to the 2015 report of the Colombian Ministry of Health and Social Protection (MHSP) ^7^, cerebrovascular disease has a global mortality rate of 32.8/100,000, an approximate incidence of 97.4/100,000. In addition, an estimated disability-adjusted life-years (DALYs) loss of 364.5 was reported ^8^.

In order to carry out clinical assessments when a gold standard is not available, or in order to complement assessments based on a gold standard (e.g., sensitivity, specificity, PPV), a reliability analysis can be undertaken. The reliability refers to the reproducibility or agreement in measurements of the variables for each case that was rated by different observers (i.e., inter-observer agreement), or cases rated by the same observer using different methods (i.e., intra-observer agreement). The agreement assessment can be achieved using loglinear agreement models, latent trait models of agreement and kappa-type statistic, as summarized by Nelson and Pepe ^9^.

In a previous work we evaluated the reading systems from another non-conventional perspective: we investigated the effect sizes of the reading systems on the magnitude of the clinical variables and their statistical equivalence by means of generalized estimated equations ^9^, showing no-effect of the reading systems on the related variables. In contrast, in the present work we evaluated reliability in terms of interpretation agreements, using the Fleiss’ kappa coefficient over the interpretations results, which is a more suitable clinical approach to evaluate the interchangeability of the evaluated devices in a clinical context. 

Although other studies have evaluated the potential equivalence between diagnostic workstations and mobile tablet computers ^10^ ^-^ ^15^, the approach that we used to perform the interpretations in a more realistic environment, along with the assessment process, and the variables evaluated, are the principal differences between our study and previous the ones; which were not set to rule out brain CT contraindications prior to intravenous thrombolysis based on image contraindications or ASPECTS scores.

## Materials and Methods

The Institutional Review Board (IRB) of our institution approved this retrospective study, and informed consent was not required. This study used an observational cross-sectional and retrospective setting to describe and to compare diagnostics test. A repeated measurements design with 1,452 interpretations were used (121 cases, 6 radiologists, and 2 reading systems. The cases were brain CT examinations acquired using a General Electric LightSpeed 64 slice CT scanner (General Electric Healthcare, GE Medical Systems, Milwaukee, WI, USA), and stored in a PACS system. Cases included brain CT images of adult males and females who arrived at our emergency room between 2013 and 2016, with symptoms of acute stroke, in whom the stroke code was activated, and the brain CT was performed as part of his stroke management. Cases were selected at random basis without repetition from the stroke database by a doctor who was no part of observers. Cases with image artifacts were excluded. Interpretations were performed by three neuroradiologists with more than ten years of experience, two with more than four years of experience, and one neuroradiology fellow. Interpretations were completed by all radiologists over all cases using two different reading systems: 

1) the routine system for CT readings in our hospital, which consist of a medical grayscale display E-2620 BARCO (BARCO N.V, Kortrijk, Belgium), and a viewer software Agfa IMPAX 6.5 (AGFA HealthCare, Mortsel, Belgium), hereafter referred to as MEDICAL-IMPAX; and, 

2) a mobile option, which consist of an Apple iPad Pro 9.7 MLMN2CL/A (Apple Inc., Cupertino, CA, USA) with a “retina” display, and a viewer software Agfa XERO Viewer 3.0 (Agfa HealthCare, Mortsel, Belgium), hereafter referred to as TABLET-XERO. The two-reading software provided image manipulation tools to adjust the window/level, the zoom, and the multiplanar reformation presentation. These tools were available for all images and could be used at the observer’s discretion to improve image interpretations. The initial display used the default image window setting (WW= 174 and WL= 55), but radiologists were free to select another window, such as a cerebral or a stroke window (WW= 80 and WL= 40 or WW= 40 and WL= 40, respectively).

We were interested in evaluating mobile solutions to be used for telestroke at the hospital. By means of real clinical scenarios that included a complete clinical background, specific neurological symptoms and the time of symptoms onset, we designed this study to perform image interpretations as closely and realistically as occur in routine clinical practice, using the first, emergency brain CT scan. We used the same radiologists who routinely read brain CT images in the hospital (i.e., neuroradiologists and neuroradiology fellows), and the same clinical information provided to radiologists in clinical practice (e.g., admission diagnosis, neurological symptoms, age and sex). In addition, the same interpretation process was conducted, in which the type of stroke (i.e., hemorrhagic lesion, acute ischemic lesion, chronic ischemic lesion, or without lesion) was classified. Furthermore, according to the selected lesion type, the radiologist classified other variables: the presence of any imaging contraindication to the t-PA administration (e.g., intraaxial neoplasm, arteriovenous malformation, aneurysm, hemorrhagic transformation of an ischemic infarct, hypodensity >1/3 of the vascular distribution of the middle cerebral artery); confidence in the presence of the hyperdense middle cerebral artery sign (HMCA); and the ASPECTS score (ranging from 0-10), which is a method for assessing the size of an infarct on CT scans in patients with acute stroke. For evaluation purposes, the ASPECTS score was dichotomized (0-6 and 7-10).

The radiologists were blinded to the patient and examination identification, to the original interpretation, and to the type of lesion. The data collection was performed using a web-based form, and interpretations were stored in a MySQL database (Oracle Corporation, Redwood City, CA, USA). This software presents the patient cases to be interpreted at random and guides the radiologists to complete the report, assuring integrity and completeness of data, therefore we have no missing data.

There were at least a five-month interval between the readings from the same patient by the same radiologist using the compared systems. This study was a counterbalanced study for the reading systems used by each radiologists and cases were presented at random.

To further evaluation of diagnostic power, by receiver operating characteristic (ROC) curves, we used the table proposed by Obuchowski ^16^, in order to determine the sample size. We adopted the following criteria: a) six observers, b) moderate variability between radiologists and high accuracy of the diagnostic examination, c) moderate differences among AUCs (i.e., 0.1) and d) a 2:1 ratio between malignant and benign cases. Using these criteria, 50 cases were required. The sample size was set at 121 cases, including all the cases available in our database in order to improve accuracy.

 The readings were performed over the course of ten months in two- or four-hour sessions by each radiologist, with no time limitation for each reading.

More detailed descriptions of the sample, the observers, the reading systems and the interpretation procedures, were presented in the our previous evaluation ^17^. However, the data analysis presented in this work is completely different, as it is a reliability evaluation.

For all variables, we evaluated intra-observer agreement (i.e., agreements on interpretations when interpreting the same patient images using the two reading systems, the MEDICAL-IMPAX and TABLET-XERO), and interobserver agreement (i.e., agreements between radiologists when interpreting the same patient within a single reading system). The agreement evaluation was performed using Fleiss’ kappa coefficient ^9^. The kappa coefficients were ranked as defined by Altman ^18^: “very good”, (κ= 1 to 0.81); “good”, (κ= 0.8 to 0.61); “moderate”, (κ= 0.6 to 0.41); “fair”, (κ= 0.4 to 0.21); and “poor”, κ <0.2. For these calculations, the software STATA 13.0 (Stata Corp, College Station, TX, USA) was used.

## Results

According to the routine primary diagnostic interpretation by a neuroradiologist, the distribution of lesions in the sample was as follows: patients without acute lesions (7), hemorrhagic lesions (11), acute ischemic lesions (67), and chronic ischemic lesions (36). The ages ranged from 30-97 years, with a mean age of 70.8 years (standard deviation of 15.2). There were 59 males and 62 females.

The intra-observer agreements between the Medical-IMPAX and Tablet-XERO reading systems for the pooled radiologists are presented in Table 1. The intra-observer agreements between the Medical-IMPAX and Tablet-XERO reading systems by individual radiologists are presented in Table 2. The interobserver agreements by reading system for the pooled radiologists are presented in Table 3.

**Table 1:** Intra-observer agreement between the Medical-IMPAX and Tablet-XERO reading systems.

Variable	Readings*	Patients	Fleiss’ Kappa†	Agreement‡
Lesion classification	1,452	121	0.63	Good
Without acute lesion			0.59	Moderate
Acute ischemic lesion			0.58	Moderate
Hemorrhagic lesion			0.89	Very Good
Presence of imaging contraindications to the t-PA administration	1,224	102	0.51	Moderate
ASPECTS classification				
Dichotomized-ASPECTS (0-6; 7-10)	828	69	0.65	Good
ASPECTS 1-3			0.66	Good
ASPECTS 4-7			0.55	Moderate
ASPECTS 8-9			0.43	Moderate
ASPECTS 10			0.68	Good

* Readings were performed over two systems by six observers.

† All values significant (*p* <0.001).

‡ As defined by Altman (18).

**Table 2:** Intra-observer agreement between the Medical-IMPAX and Tablet-XERO reading systems by radiologist.

Variable/Radiologist	Fleiss’ Kappa*	Agreement^**†**^
Lesion classification		
Neuroradiologists	0.73	Good
Neuroradiologists	0.57	Moderate
Neuroradiologists	0.55	Moderate
Neuroradiologists	0.77	Good
Neuroradiologists	0.49	Moderate
Neuroradiology fellow	0.67	Good
Without acute lesion		
Neuroradiologists	0.70	Good
Neuroradiologists	0.52	Moderate
Neuroradiologists	0.53	Moderate
Neuroradiologists	0.73	Good
Neuroradiologists	0.42	Moderate
Neuroradiology fellow	0.62	Good
Acute ischemic lesion		
Neuroradiologists	0.70	Good
Neuroradiologists	0.53	Moderate
Neuroradiologists	0.47	Moderate
Neuroradiologists	0.75	Good
Neuroradiologists	0.41	Moderate
Neuroradiology fellow	0.62	Good
Hemorrhagic lesion		
Neuroradiologists	0.89	Very Good
Neuroradiologists	0.89	Very Good
Neuroradiologists	0.82	Very Good
Neuroradiologists	0.95	Very Good
Neuroradiologists	0.87	Very Good
Neuroradiology fellow	0.95	Very Good

* Each agreement was calculated from 242 readings (121 cases by 2 reading systems). All values significant (*p* <0.001).

† As defined by Altman (18).

**Table 3:** Interobserver agreement by reading system.

Variable	Reading system	Fleiss’ Kappa*	Agreement
Lesion classification	Medical-IMPAX	0.56	Moderate
	Tablet-XERO	0.56	Moderate
Without acute lesion	Medical-IMPAX	0.50	Moderate
	Tablet-XERO	0.52	Moderate
Acute ischemic lesion	Medical-IMPAX	0.50	Moderate
	Tablet-XERO	0.51	Moderate
Hemorrhagic lesion	Medical-IMPAX	0.90	Very Good
	Tablet-XERO	0.82	Very Good
Presence of imaging contraindications to the t-PA administration	Medical-IMPAX	0.38	Fair
	Tablet-XERO	0.33	Fair
ASPECTS classification			
Dichotomized-ASPECTS (0-6;7-10)	Medical-IMPAX	0.51	Moderate
	Tablet-XERO	0.48	Moderate
ASPECTS 1-3	Medical-IMPAX	0.29	Fair
	Tablet-XERO	0.30	Fair
ASPECTS 4-7	Medical-IMPAX	0.41	Moderate
	Tablet-XERO	0.41	Moderate
ASPECTS 8-9	Medical-IMPAX	0.34	Fair
	Tablet-XERO	0.28	Fair
ASPECTS 10	Medical-IMPAX	0.57	Moderate
Tablet-XERO	0.59	Moderate

* There were 726 readings for lesion classification (121cases by 6 observers); 612 readings for the presence of imaging contraindications to the t-PA administration (102 cases by 6 observers); and 414 readings for the ASPECTS classification administration (69 cases by 6 observers). All values significant (*p* <0.001).

† As defined by Altman (18).

### Agreements on the lesion classification

There was good intra-observer agreement on the lesion classification, for the pooled group of radiologists, when each patient was interpreted using both the Medical-IMPAX and the TABLET-XERO reading systems by the same observer (κ= 0.63, *p*<0.001), as shown in Table 1. Marginal agreements for this variable, for the pooled group of radiologists, were very good on hemorrhagic lesion (κ= 0.89, *p*<0.001), and moderate on both without acute lesion classification (κ= 0.59, *p*<0.001) and on acute ischemic lesion classification (κ= 0.58, *p*<0.001).

Individual radiologist’s intra-observer agreements on the lesion classification ranged from κ= 0.49-0.77 (all *p* <0.001), and agreements were ranked as good or moderate, regardless the radiologists experience (Table 2).

For individual radiologists, marginal intra-observer agreements for this variable were as follows: there was very good agreements on hemorrhagic lesion for all radiologists, κ= 0.82-0.95 (all *p* <0.001); moderate or good agreements on the “other lesion” classifications for all radiologists (regardless of radiologist experience), κ= 0.42-0.73 (all *p* <0.001) and without acute lesion classifications, κ= 0.41-0.75 (all *p* <0.001) (Table 2).

There was moderate interobserver agreements on the lesion classification (κ= 0.56, *p* <0.001), on both the Medical-IMPAX and the TABLET-XERO reading systems (Table 3). Marginal agreements for this variable were very good on hemorrhagic lesion classification for both the Medical-IMPAX and the TABLET-XERO reading systems, with κ= 0.90 (*p* <0.001) and κ= 0.82 (*p* <0.001), respectively. All the other marginal interobserver agreements on lesion classification were moderate in both the Medical-IMPAX and the TABLET-XERO reading systems, and ranged from κ= 0.50-0.52.

### Agreements on the ASPECTS score

#### Agreements on the Dichotomized-ASPECTS score

There was good intra-observer agreement on the dichotomized-ASPECTS (i.e., 0-6; 7-10), for the pooled group of radiologists, when each patient was interpreted using both the Medical-IMPAX and the TABLET-XERO reading systems (κ= 0.65, *p*<0.001), as shown in Table 1.

There was moderate interobserver agreement on the dichotomized-ASPECTS, on both the Medical-IMPAX and the TABLET-XERO reading systems, with κ= 0.51 (*p* <0.001) and κ= 0.48 (*p* <0.001), respectively (Table 3). 

#### Agreements on the categorical-ASPECTS scores

To compare our results with those of a recent study published by McLaughlin et al. ^10^, in addition to the dichotomized ASPECTS, we calculated the agreements on the ASPECTS scores with the same categories defined in that study, i.e., ASPECTS scores grouped into four categories (1-3, 4-7, 8-9, and 10). 

There was good intra-observer agreements on the categorical-ASPECTS scores, for the pooled group of radiologists, when each patient was interpreted using both the Medical-IMPAX and the TABLET-XERO reading systems for 1-3 and 10 categories (κ= 0.66 and κ= 0.68 respectively, both *p*<0.001), as shown in Table 1.

There was fair interobserver agreement on the categorical-ASPECTS scores, on both the Medical-IMPAX and the TABLET-XERO reading systems for categories 1-3 and 8-9, but there was moderate agreements on both the Medical-IMPAX and the TABLET-XERO reading systems for categories 4-7 and 10 (Table 3), having both reading systems very similar kappa values in each category.

#### Agreements on the presence of imaging contraindications to the t-PA administration

There was moderate intra-observer agreement on the presence of imaging contraindications to the t-PA administration, for the pooled group of radiologists, when each patient was interpreted using both the Medical-IMPAX and the TABLET-XERO reading systems (κ= 0.51, *p*<0.001), as shown in Table 1.

There was fair interobserver agreement on the presence of imaging contraindications to the t-PA administration, on both the Medical-IMPAX and the TABLET-XERO reading systems, with κ= 0.38 (*p* <0.001) and κ= 0.33 (*p* <0.001), respectively (Table 3).

## Discussion

Good intra-observer agreement on the lesion classification was observed, with marginal agreements ranked as very good on hemorrhagic lesion and moderate on the other type of classification lesions. This suggests that when any radiologist interprets the same patient images using any of the two reading systems, the MEDICAL-IMPAX or TABLET-XERO, there are no differences in the initial patient’s outcome. This is remarkable in the detection of patients with hemorrhagic lesion for whom the t-PA administration is not appropriate. This result was independent of the radiologists’ experience. In our study, observers where all neuroradiologists with different levels of experience, and training periods, nevertheless in our hospital, all interpretation are performed only by neuroradiologists with more than four years of experience. Interobserver agreements for a single reading system (i.e., when all radiologists uses the same reading system), were the same on each classification variable (moderate or very good), suggesting that there are no-superiority of any reading system over the other.

Ours results on hemorrhagic lesions correlate with results of prior studies, in which high agreements and accuracy were found for this stroke subcategory ^10^ ^-^ ^12^. This is crucial given that this is the most important contraindication for t-PA administration. In the same sense, patients with ASPECTS scores ≤6 are not considered eligible for this treatment, the reason why we evaluated the ASPECTS score in categories 0-6 and 7-10. Good intra-observer agreement on the dichotomized-ASPECTS were observed. Interobserver agreements on the dichotomized-ASPECTS score were all ranked as moderate agreement, regardless of the reading system, suggesting again that there are no-superiority of any reading system over the other on this clinical variable.

Moderate intra-observer agreement on the presence of imaging contraindications to the t-PA administration, other than hemorrhagic lesions or patients with ASPECTS ≤6, was observed. Although fair interobserver agreement was observed using the TABLET-XERO, the same result was observed for the Medical-IMPAX.

Besides, interobserver agreements on these variables (Table 3) were all ranked in the same agreement category, regardless of the reading system, suggesting again that there are no-superiority of any reading system over the other.

One limitation of this study is that reliability on the detection of the presence of a hyperdense middle cerebral artery (HMCA) was not possible to be evaluated, as the number of cases with scores assigned by the six radiologists on the two reading systems (i.e., 12 scores assigned to each case) was very low to perform a reliable analysis of this variable. Interpretations on the Medical-IMPAX system were performed using the same setup as in clinical routine interpretations, including the illumination conditions. In contrast, interpretations on the TABLET-XERO were performed using non-controlled illumination conditions; nevertheless, this is a more realistic telestroke context, in which a radiologist must to read emergency images at any place.

The study of Park *et al.* ^13^, found moderate intra-observer agreement (κ= 0.597) on the detection of intracranial hemorrhages, while in our study, agreement on the detection of hemorrhagic lesions was very good (κ= 0.89). In our study, we observed very good interobserver agreements on the detection of hemorrhagic lesion, with kappa values of 0.9 for the reference display and 0.82 for the tablet; however, in their study, these values were not presented. Differences in the kappa values may arise from the sample selection; in their study, subtle intracranial hemorrhages were included, while in our study, we included subarachnoid hemorrhage and intracranial hemorrhage, but not all being subtle hemorrhages. In addition, their study was based on reading of five emergency physicians, whereas in our study they were only performed by neuroradiologists.

The study of McLaughlin *et al*. ^10^, used the same kind of tablet as in our study. They evaluated the ASPECTS interobserver agreement for individual observers, grouping the ASPECTS scores into four categories (1-3, 4-7, 8-9, and 10). Substantial interobserver agreements were noted for the neuroradiologists and fair agreements for junior radiologists. They used two neuroradiologists in consensus to evaluate agreement using the tablet, but they did not calculate agreements for individual neuroradiologists neither on tablet, nor on the reference display. In addition, they did not calculate intra-observer agreements. To compare with this study, in addition to the dichotomized ASPECTS, we calculated the agreements on the categorical-ASPECTS scores with the same categories for the overall group of radiologists, presented in Table 1. Values for individual radiologists are not presented as with few patients by radiologist in each category, the kappa values where not reliable (all *p* >0.05). In our study, all intra-observer categories were ranked as good or moderate, with kappa values from 0.43 to 0.68. As in the study of McLaughlin, highest kappa values were observed for categories 1-3 and 10 and were lowest for categories 4-7 and 8-9. 

## Conclusion

There was no-superiority of any reading system over the other, on the agreements on the clinical variables evaluated, as was in our previous evaluation, in which all the related variables were statistically equivalent (at the selected threshold of 10%), when using both Medical-IMPAX and Tablet-XERO the reading systems. This is a relevant fact which provides evidence that the Medical-IMPAX and Tablet-XERO reading systems may be interchangeable, without a loss of reliability, when neuroradiologists uses the mobile solution for interpreting brain CT images of patients with symptoms of acute stroke. Hence, increasing efficiency, supply and availability of telestroke services to underserved populations.

In the statistical design of this study, the radiologist and the readings systems were fixed factors, as they were not selected at random; therefore, our results only apply to them. Nevertheless, as neuroradiologists are very specialized radiologists, we expect that our results may be generalized to other neuroradiologists. Similarly, the readings systems in radiology must be DICOM-compliant, which allows us to generalize our results to other reading software or medical displays. In contrast, the tablet display may be very different, and our results apply only to them using “retina” displays.

In our study, brain CT readings using a tablet were performed over a Wi-Fi connection; for a more realistic approach in our country, where public Wi-Fi is not always available, an evaluation of the cost and time for image transfer over mobile telephone networks such as the 4G network is necessary. 
