# Supplementary material for: Mobile device for thrombolysis decisions for telestroke
Source: Colomb Med (Cali). 2018 Dec 30;49(4):254–60. doi: 10.25100/cm.v49i4.3921 (PMC6342084; doi:10.25100/cm.v49i4.3921)
Supplement: Related texts

# Original article: Dispositivo móvil para decisiones de trombólisis mediante Telestroke

## Introducción

Los pacientes que llegan a la sala de urgencias con síntomas de accidente cerebrovascular agudo (ACV) son diagnosticados y tratados utilizando un protocolo estandarizado y bien establecido llamado *código ACV*, para el cual se realizan varias tareas. Uno de los principales resultados para este grupo de pacientes es determinar si se debe tratar al paciente administrando activador del plasminógeno tisular (AP-t) o si se debe realizar una trombectomía endovascular. Como parte de este protocolo, se realiza un examen por tomografía axial computarizada (TAC) de cráneo sin contraste ^1^^,^^2^, la cual es interpretada por neurorradiólogos experimentados que, en primer lugar, deben establecer si no existen contraindicaciones para la administración de AP-t con base en las imágenes suministradas. Adicionalmente, además de la experiencia del neurólogo, se determina el tratamiento para el paciente, el cual es crucial en su evolución.

Durante la interpretación de la TAC de cráneo simple se debe clasificar la lesión del paciente como una de las siguientes: lesión hemorrágica, ACV agudo, ACV crónico o sin lesión aguda. Una lesión hemorrágica es una contraindicación para la administración de AP-t; otras contraindicaciones pueden aparecer si hay ciertas señales, como por ejemplo, la presencia de neoplasia intra-axial, neoplasia intracraneal, malformación arteriovenosa, aneurisma o transformación hemorrágica de un infarto isquémico. Adicionalmente si el *Alberta Stroke Program Early CT Score* (ASPECTS) ^3^ del paciente, el cual se utiliza para evaluar el tamaño del infarto en las imágenes de TAC de cráneo simple, es menor que o igual a seis, el paciente no es elegible para la administración de AP-t ^1^^,^^2^^,^^4^^,^^5^, ya que hay un alto riesgo de infarto maligno de la arteria cerebral media después de la administración de AP-t ^6^. Por estos motivos, la interpretación de la TAC de cráneo simple de pacientes con signos de ACV agudo en nuestro hospital es realizada por neurorradiólogos con experiencia significativa en ACVs. Sin embargo, la mayoría de hospitales de nuestro país no cuenta con neurorradiólogos en absoluto. Similarmente, nuestra ubicación, que es un centro primario de ACV acreditado por la Comisión Conjunta Internacional (JCI, por sus siglas en inglés), con capacidades de trombectomía endovascular, no cuenta con suficientes neurorradiólogos para apoyar una red de teleACV. Con el fin de aumentar la oferta y disponibilidad de neurorradiólogos, se pueden desarrollar soluciones móviles utilizando computadores tableta.

El objetivo de este estudio era evaluar la confiabilidad de lecturas de imágenes de TAC de cráneo simple realizadas mediante una estación de trabajo diagnóstico principal y un computador tableta en el contexto de la red colombiana de teleACV de urgencia. Según un informe del Ministerio de Salud y Protección Social de Colombia (MSPS) publicado en el año 2015 ^7^, la enfermedad cerebrovascular tiene una tasa de mortalidad mundial de 32.8/100,000 y una incidencia aproximada de 97,4/100.000. Adicionalmente, se reportó una pérdida aproximada de años de vida ajustados por discapacidad (AVAD) de 364.5 ^8^.

Con el fin de realizar evaluaciones clínicas cuando no se cuenta con un patrón de referencia, o con el fin de complementar evaluaciones basadas en un patrón de referencia (por ejemplo, sensibilidad, especificidad, VPP), se puede realizar un análisis de confiabilidad. La confiabilidad se refiere a la reproducibilidad o concordancia en las medidas de las variables para cada caso que fue evaluado por observadores diferentes (concordancia interobservadores), o casos evaluados por el mismo observador utilizando métodos diferentes (concordancia intraobservadores). La evaluación de la concordancia se puede lograr utilizando modelos logarítmicos lineales de concordancia y estadísticos kappa, según fueron resumidos por Nelson y Pepe ^9^.

En un trabajo anterior evaluamos los sistemas de lectura desde otra perspectiva no convencional: investigamos los tamaños del efecto de los sistemas de lectura sobre la magnitud de las variables clínicas y su equivalencia estadística mediante ecuaciones de estimación generalizadas ^9^, mostrando que los sistemas de lectura no tienen efectos sobre las variables relacionadas. En contraste, en este trabajo evaluamos la confiabilidad en términos de concordancias de interpretación, utilizando el coeficiente kappa de Fleiss sobre los resultados de las interpretaciones, el cual es un acercamiento clínico más apropiado para evaluar la intercambiabilidad de los dispositivos evaluados en un contexto clínico. 

Si bien otros estudios han evaluado la equivalencia potencial entre estaciones de trabajo de diagnóstico y computadores tableta ^10^^-^^15^, el enfoque que utilizamos para realizar las interpretaciones en un entorno más realista, junto con el proceso de evaluación y las variables evaluadas, son las principales diferencias entre nuestro estudio y los estudios anteriores, los cuales no se realizaron para descartar contraindicaciones en TAC de cráneo simples antes de la trombólisis intravenosa con base en las contraindicaciones en las imágenes o en los puntajes ASPECTS.

## Materiales y Métodos

El Comité de Ética de nuestra institución aprobó este estudio retrospectivo y no se requería de un consentimiento informado. Este estudio utilizó un ajuste transversal y retrospectivo de observación para describir y comparar pruebas diagnósticas. Se utilizó un diseño de mediciones repetidas con 1,452 interpretaciones (121 casos, 6 radiólogos y 2 sistemas de lectura). Los casos fueron exámenes de TAC de cráneo simples utilizando un TAC Genera Electric LigthSpeed de 64 rebanadas (General Electric Healthcare, GE Médico Systems, Milwaukee, WI, EE. UU.), almacenados en el sistema PACS. Los casos incluyeron imágenes de TAC de cráneo simple de hombres y mujeres adultos que llegaron a la sala de urgencias entre el 2013 y el 2016 con síntomas de ACV agudo, en quienes se activó el código ACV y se realizó el TAC de cráneo simple como parte del manejo de su ACV. Los casos fueron seleccionados aleatoriamente, sin repetición, de la base de datos de ACV por un doctor que no fue parte de los observadores. Los casos con artefactos en la imagen fueron excluidos. Tres neurorradiólogos con más de diez años de experiencia, dos con más de cuatro años de experiencia y un becario de neurorradiología realizaron las interpretaciones. Todos los radiólogos realizaron las interpretaciones de todos los casos utilizando dos sistemas de lectura diferentes: 

1) el sistema de rutina para las lecturas de TAC en nuestro hospital, el cual consiste en una pantalla médica en escala de grises E-2620 BARCO (BARCO N.V, Cortrique, Bélgica) y el software de visualización Agfa IMPAX 6.5 (AGFA HealthCare, Mortsel, Bélgica), en adelante llamado MEDICAL-IMPAX y 

2) una opción móvil, que consiste en un iPad Pro de Apple 9.7 MLMN2CL/A (Apple Inc., Cupertino, CA, EE. UU.) con una pantalla ‘retina’ y un software de visualización Agfa XERO Viewer 3.0 (Agfa HealthCare, Mortsel, Bélgica), en adelante llamado Tableta-XERO. Los dos softwares de lectura cuentan con herramientas de manipulación de la imagen para ajustar la ventana/nivel, el zoom y la presentación de la reconstrucción multiplanar. Estas herramientas estaban disponibles para todas las imágenes y podían ser utilizadas a discreción del observador para mejorar la interpretación de las imágenes. La visualización inicial utilizó la configuración de ventana por defecto de la imagen (WW= 174 y WL= 55), pero los radiólogos tenían la libertad de seleccionar otra ventana, como por ejemplo la ventana cerebral o de ACV (WW= 80 y WL= 40 o WW= 40 y WL= 40, respectivamente).

Estábamos interesados en evaluar las soluciones móviles a ser utilizadas para teleACV en el hospital. A través de escenarios médicos reales que incluyeron un historial médico completo, síntomas neurológicos específicos y el momento de aparición de los síntomas, diseñamos este estudio para realizar la interpretación de las imágenes tan cercana y realísticamente a como ocurren durante las prácticas clínicas de rutina, utilizando el primer TAC de cráneo simple de urgencia. Utilizamos los mismos radiólogos que, rutinariamente, leen las imágenes de TAC de cráneo simple en el hospital (neurorradiólogos y becarios en neurorradiología), y la misma información clínica suministrada a los radiólogos en la práctica clínica (diagnóstico de admisión, síntomas neurológicos, edad y género). Adicionalmente, se realizó el mismo proceso de interpretación, en el que se determina el tipo de ACV (lesión hemorrágica, lesión isquémica aguda, lesión isquémica crónica o sin lesión). Adicionalmente, dependiendo del tipo de lesión seleccionada, el radiólogo clasificó otras variables: la presencia de contraindicaciones en la imagen a la administración de AP-t (neoplasia intra-axial, malformación arteriovenosa, aneurisma, transformación hemorrágica de un infarto isquémico, hipodensidad >1/3 de la distribución vascular de la arteria cerebral media); confianza en la presencia de Arteria Cerebral Media Hiperdensa (ACMH); y el puntaje ASPECTS (de 0 a 10), el cual es un método para evaluar el tamaño de un infarto en las imágenes de la TAC de pacientes con ACV agudo. El puntaje ASPECTS fue dicotomizado con fines de evaluación (0-6 y 7-10).

Los radiólogos no tuvieron acceso a la identificación del paciente ni del examen, a la interpretación original ni al tipo de lesión. La información fue recolectada utilizando un formulario basado en la web y las interpretaciones fueron almacenadas en una base de datos MySQL (Oracle Corporation, Redwood City, CA, EE. UU.). Este software presenta los casos de pacientes a ser interpretados aleatoriamente y guía a los radiólogos en el diligenciamiento del informe, garantizando la integridad de los datos; por lo tanto, no hay datos faltantes.

Hubo un intervalo de al menos cinco meses entre las lecturas del mismo paciente por el mismo radiólogo utilizando los sistemas comparados. Este estudio fue un estudio equilibrado para los sistemas de lectura utilizados por cada uno de los radiólogos y los casos fueron presentados aleatoriamente.

Para una mayor evaluación de la potencia de diagnóstico mediante curvas de Característica Operativa del Receptor (ROC, por sus siglas en inglés), utilizamos la tabla propuesta por Obuchowski ^16^ para determinar el tamaño de la muestra. Adoptamos los siguientes criterios: a) seis observadores, b) variabilidad moderada entre radiólogos y alta precisión del examen diagnóstico, c) diferencias moderadas entre AUC (0.1) y d) una relación de 2:1 entre casos malignos y benignos. fueron necesarios incluir 50 casos utilizando estos criterios. Se estableció un tamaño de muestra de 121 casos, incluyendo todos los casos disponibles en nuestra base de datos, con el fin de aumentar la precisión.

Las lecturas fueron realizadas en el curso de diez meses, en sesiones de dos o cuatro horas para cada radiólogo, sin límites de tiempo para cada lectura.

Nuestra evaluación anterior ^17^ presentó descripciones más detalladas de la muestra, los observadores, los sistemas de lectura y los procedimientos de interpretación. Sin embargo, el análisis de datos presentado en este artículo es completamente diferente, ya que se trata de una evaluación de confiabilidad.

Para todas las variables evaluamos la concordancia intraobservadores (concordancias en las interpretaciones al interpretar las imágenes del mismo paciente utilizando los dos sistemas de lectura, el MEDICAL-IMPAX y el Tableta-XERO) y la concordancia interobservadores (concordancia entre los radiólogos al interpretar al mismo paciente dentro de un único sistema de lectura). La evaluación de concordancia se realizó utilizando el coeficiente kappa de Fleiss ^9^. Los coeficientes kappa fueron clasificados según lo definido por Altman ^18^: “muy bueno”, (κ= 1.0 a 0.81); “bueno”, (κ= 0.8 a 0.61); “moderado”, (κ= 0.6 a 0.41); “regular”, (κ= 0.4 a 0.21); y “pobre”, κ <0.2. Para estos cálculos se utilizó el software STATA 13.0 (Stata Corp, College Station, TX, EE. UU.).

## Resultados

Según la interpretación diagnóstica primaria de rutina realizada por un neurorradiólogo, la distribución de las lesiones en la muestra fue la siguiente: pacientes sin lesiones agudas (7), lesiones hemorrágicas (11), lesiones isquémicas agudas (67) y lesiones isquémicas crónicas (36). Las edades estaban entre los 30 y los 97 años, con una edad media de 70.8 años (desviación estándar de 15,2). Había 59 hombres y 62 mujeres.

La Tabla 1 presenta las concordancias intraobservadores entre los sistemas de lectura Médico-IMPAX y Tableta-Xero para los radiólogos agrupados. La Tabla 2 presenta las concordancias intraobservadores entre los sistemas de lectura Médico-IMPAX y Tableta-XERO para los radiólogos individuales. La Tabla 3 presenta las concordancias interobservadores por sistema de lectura para los radiólogos agrupados.

**Table 1:** Concordancia intraobservador entre los sistemas de lectura Médico-IMPAX y Tableta-XERO.

Variable	Lecturas*	Pacientes	Fleiss Kappa†	Concordancia‡
Clasificación de la lesión	1,452	121	0.63	Buena
Sin lesiones agudas			0.59	Moderada
Lesión isquémica aguda			0.58	Moderada
Lesión hemorrágica			0.89	Muy buena
**Presencia de contraindicaciones por imágenes para la para la administración de AP-t**	1,224	102	0.51	Moderada
**Clasificación del ASPECTS**				
ASPECTS dicotomizado (0-6; 7-10)	828	69	0.65	Buena
ASPECTS 1-3			0.66	Buena
ASPECTS 4-7			0.55	Moderada
ASPECTS 8-9			0.43	Moderada
ASPECTS 10			0.68	Buena

* Las lecturas fueron realizadas en dos sistemas de lectura por seis observadores.

† Todos los valores fueron significativos (p < 0.001).

‡ Como lo define Altman (18).

**Table 2:** Concordancia intraobservador entre los sistemas de lectura Médico-IMPAX y Tableta-XERO por radiólogo.

Variable/Radiólogo	Fleiss Kappa*	Concordancia†
Clasificación de la lesión		
Neurorradiólogo	0.73	Buena
Neurorradiólogo	0.57	Moderada
Neurorradiólogo	0.55	Moderada
Neurorradiólogo	0.77	Buena
Neurorradiólogo	0.49	Moderada
Fellow de neurorradiología	0.67	Buena
Sin lesiones agudas		
Neurorradiólogo	0.70	Buena
Neurorradiólogo	0.52	Moderada
Neurorradiólogo	0.53	Moderada
Neurorradiólogo	0.73	Buena
Neurorradiólogo	0.42	Moderada
Fellow de neurorradiología	0.62	Buena
Lesión isquémica aguda		
Neurorradiólogo	0.70	Buena
Neurorradiólogo	0.53	Moderada
Neurorradiólogo	0.47	Moderada
Neurorradiólogo	0.75	Buena
Neurorradiólogo	0.41	Moderada
Fellow de neurorradiología	0.62	Buena
Lesión hemorrágica		
Neurorradiólogo	0.89	Muy buena
Neurorradiólogo	0.89	Muy buena
Neurorradiólogo	0.82	Muy buena
Neurorradiólogo	0.95	Muy buena
Neurorradiólogo	0.87	Muy buena
Fellow de neurorradiología	0.95	Muy buena

* Cada concordancia se calculó a partir de 242 lecturas (121 casos por 2 sistemas de lectura). Todos los valores fueron significativos (p <0.001).

† Como lo define Altman (18).

**Table 3:** Concordancia interobservador por sistema de lectura.

Variable	Sistema de lectura	Fleiss Kappa*	Concordancia†
Clasificación de la lesión	Médico-IMPAX	0.56	Moderada
	Tableta-XERO	0.56	Moderada
Sin lesiones agudas	Médico-IMPAX	0.50	Moderada
	Tableta-XERO	0.52	Moderada
Lesión isquémica aguda	Médico-IMPAX	0.50	Moderada
	Tableta-XERO	0.51	Moderada
Lesión hemorrágica	Médico-IMPAX	0.90	Muy buena
	Tableta-XERO	0.82	Muy buena
Presencia de contraindicaciones por imágenes para la para la administración de AP-t	Médico-IMPAX	0.38	Regular	Tableta-XERO	0.33	Regular
Clasificación del ASPECTS			
ASPECTS dicotomizado (0-6; 7-10)	Médico-IMPAX	0.51	Moderada
	Tableta-XERO	0.48	Moderada
ASPECTS 1-3	Médico-IMPAX	0.29	Regular
	Tableta-XERO	0.30	Regular
ASPECTS 4-7	Médico-IMPAX	0.41	Moderada
	Tableta-XERO	0.41	Moderada
ASPECTS 8-9	Médico-IMPAX	0.34	Regular
	Tableta-XERO	0.28	Regular
ASPECTS 10	Médico-IMPAX	0.57	Moderada
Tableta-XERO	0.59	Moderada

* 726 lecturas para la Clasificación de la lesión (121 casos por 6 observadores); 612 lecturas para las Contraindicaciones de trombólisis por imágenes (102 casos por 6 observadores); y 414 lecturas para la Clasificación del ASPECTS (69 casos por 6 observadores). Todos los valores fueron significativos (p <0.001).

† Como lo define Altman (18).

### Concordancias en la clasificación de la lesión

Hubo una buena concordancia intraobservadores en la clasificación de la lesión para os radiólogos agrupados, en la que cada paciente fue interpretado utilizando tanto el sistema de lectura Médico-IMPAX como el Tableta-XERO por el mismo observador (κ= 0.63 *p*<0.001), tal y como se muestra en la Tabla 1. Las concordancias marginales para esta variable, para los radiólogos agrupados, fue muy buena para la lesión hemorrágica (κ= 0.89, *p*<0.001) y moderada para la clasificación de ausencia de lesión aguda (κ= 0.59, *p*<0.001) y para la clasificación de lesión isquémica aguda (κ= 0.58, *p*<0.001).

Las concordancias intraobservadores para los radiólogos individuales en la clasificación de la lesión varío de κ= 0.49-0.77 (todos *p* <0.001) y las concordancias fueron clasificadas como buenas o moderadas, independientemente de la experiencia de los radiólogos (Tabla 2).

Para los radiólogos individuales, las concordancias marginales intraobservadores para esta variable fueron las siguientes: hubo concordancias muy buenas en lesión hemorrágica para todos los radiólogos, κ= 0.82-0.95 (todos *p* <0.001); concordancias moderadas o buenas en las clasificaciones de “otras lesiones” para todos los radiólogos (independientemente de la experiencia del radiólogo), κ= 0.42-0.73 (todos *p* <0.001) y sin clasificaciones de lesiones agudas κ= 0.41-0.75 (todos *p* <0.001) (Tabla 2).

Hubo concordancias interobservadores moderadas en la clasificación de la lesión (κ= 0.56, *p* <0.001) tanto para el sistema de lectura Médico-IMPAX como para el Tableta-XERO (Tabla 3). Las concordancias marginales para esta variable fueron muy buenas en la clasificación de lesión hemorrágica tanto para el sistema de lectura Médico-IMPAX como para el Tableta-XERO, con κ= 0.90 (*p* <0.001) y κ= 0.82 (*p* <0.001), respectivamente. Todas las otras concordancias interobservadores marginales en la clasificación de la lesión fueron moderadas tanto para el sistema de lectura Médico-IMPAX como para el Tableta-XERO, y estuvieron en un rango de κ= 0.50 a 0.52.

### Concordancia en el puntaje ASPECTS

#### Concordancia en el puntaje ASPECTS dicotomizado

Hubo una buena concordancia intraobservadores en el ASPECTS dicotomizado (0-6; 7-10) para los radiólogos agrupados, en la que cada paciente fue interpretado utilizando tanto el sistema de lectura Médico-IMPAX como el Tableta-XERO (κ= 0.65, *p*<0.001), tal y como se muestra en la Tabla 1.

Hubo una concordancia interobservadores moderada en el ASPECTS dicotomizado tanto para el sistema de lectura Médico-IMPAX como para el Tableta-XERO, con κ= 0.51 (*p* <0.001) y κ= 0.48 (*p* < 0.001), respectivamente (Tabla 3). 

#### Concordancia en los puntajes ASPECTS categorizados

Para comparar nuestros resultados con los de un estudio reciente publicado por McLaughlin *et al*.^10^, además del ASPECTS dicotomizado, calculamos las concordancias de los puntajes ASPECTS con las mismas categorías definidas en ese estudio, es decir, puntajes ASPECTS agrupados en cuatro categorías (1-3, 4-7, 8-9 y 10). 

Hubo una buena concordancia intraobservadores en los puntajes ASPECTS categorizados para los radiólogos agrupados, en el que cada paciente fue interpretado utilizando tanto el sistema de lectura Médico-IMPAX como el Tableta-XERO para las categorías 1-3 y 10 (κ= 0.66 y κ= 0.68 respectivamente, ambos *p*<0.001), tal y como se muestra en la Tabla 1.

Hubo una concordancia interobservadores regular en los puntajes ASPECTS categorizados tanto para el sistema de lectura Médico-IMPAX como para el Tableta-XERO para las categorías 1-3 y 8-9, pero hubo concordancias moderadas tanto para el sistema de lectura Médico-IMPAX como para el Tableta-XERO para las categorías 4-7 y 10 (Tabla 3), con ambos sistemas de lectura presentando valores kappa similares en cada categoría.

#### Concordancia sobre la presencia de contraindicaciones en imágenes para la administración de AP-t

Hubo una concordancia intraobservadores moderada en la presencia de contraindicaciones en imágenes para la administración de AP-t para los radiólogos agrupados, en la que cada paciente fue interpretado utilizando tanto el sistema de lectura Médico-IMPAX como el Tableta-XERO (κ= 0.51, *p*<0.001), tal y como se muestra en la Tabla 1.

Hubo una concordancia interobservadores regular en la presencia de contraindicaciones en imágenes para la administración de AP-t tanto para el sistema de lectura Médico-IMPAX como para el Tableta-XERO, con κ= 0.38 (*p* <0.001) y κ= 0.33 (*p* <0.001), respectivamente (Tabla 3).

## Discusión

Se observó una buena concordancia intraobservadores en la clasificación de la lesión, con las concordancias marginales clasificadas como muy buenas para lesión hemorrágica y moderadas para otros tipos de clasificación de lesiones. Esto sugiere que, cuando cualquier radiólogo interpreta las imágenes del mismo paciente utilizando cualquiera de los dos sistemas de lectura, el MEDICAL-IMPAX o el Tableta-XERO, no existen diferencias en los resultados iniciales de los pacientes. Esto es notable en la detección de pacientes con lesiones hemorrágicas para quienes la administración de AP-t no es apropiada. Este resultado fue independiente de la experiencia de los radiólogos. En nuestro estudio, todos los observadores eran neurorradiólogos con diferentes niveles de experiencia y periodos de entrenamiento; no obstante, toda la interpretación en nuestro hospital es realizada únicamente por neurorradiólogos con más de cuatro años de experiencia. Las concordancias interobservadores para un único sistema de lectura (cuando todos los radiólogos utilizaron el mismo sistema de lectura) fueron las mismas en cada variable de clasificación (moderada o muy buena), lo que sugiere que no existe superioridad de un sistema por encima del otro.

Nuestros resultados en lesiones hemorrágicas se correlacionan con los resultados de estudios anteriores, en los que se encontraron altas concordancias y precisión para esta subcategoría de ACV ^10^^-^^12^. Esto es crucial, dado que es la contraindicación más importante para la administración de AP-t. Del mismo modo, los pacientes con puntajes ASPECTS ≤6 no son considerados como elegibles para este tratamiento, razón por la cual evaluamos los puntajes ASPECTS en las categorías 0-6 y de 7-10. Se observó una buena concordancia intraobservadores en el ASPECTS dicotomizado. Todas las concordancias interobservadores en los ASPECTS dicotomizados fueron calificadas como concordancia moderada, independientemente del sistema de lectura, sugiriendo una vez más que no existe superioridad de uno de los sistemas de lectura sobre el otro en esta variable clínica.

Se observó una concordancia intraobservadores moderada en la presencia de contraindicaciones en imágenes para la administración de AP-t, excepto por lesiones hemorrágicas o pacientes con ASPECTS ≤6. Si bien se observó una concordancia interobservadores regular utilizando Tableta-XERO, se observó el mismo resultado para el Médico-IMPAX.

Por otra parte, las concordancias interobservadores de estas variables (Tabla 3) fueron clasificadas en la misma categoría de concordancia independientemente del sistema de lectura, lo que nuevamente sugiere que no existe superioridad de un sistema por encima del otro.

Una limitación de este estudio es que no fue posible evaluar la confiabilidad en la detección de la presencia de una arteria cerebral media hiperdensa (ACMH), dado que el número de casos con puntajes asignados por los seis radiólogos en los dos sistemas de lectura (es decir, 12 puntajes asignados en cada caso) era muy bajo para realizar un análisis confiable de esta variable. La interpretación del sistema Médico-IMPEX se realizó utilizando la misma configuración que en las interpretaciones clínicas de rutina, incluyendo las condiciones de iluminación. En contraste, las interpretaciones con Tableta-XERO fueron realizadas utilizando condiciones de iluminación no controladas; no obstante, este es un contexto más realista para teleACV, en el que un radiólogo debe leer imágenes de urgencia en cualquier lugar.

El estudio realizado por Park *et al.* ^13^, encontró una concordancia intraobservadores moderada (κ= 0.597) en la detección de hemorragias intracraneales, mientras que en nuestro estudio, la concordancia en la detección de lesiones hemorrágicas fue muy buena (κ= 0.89). En nuestro estudio observamos concordancias interobservadores muy buenas en la detección de lesión hemorrágica, con valores kappa de 0.90 para la imagen de referencia y 0.82 para la tableta; sin embargo, estos valores no fueron presentados en su estudio. Las diferencias en los valores kappa puede surgir en la selección de muestras; en su estudio, se incluyeron hemorragias intracraneales sutiles, mientras que nuestro estudio incluyó hemorragia subaracnoidea y hemorragia intracraneal, pero no todas eran hemorragias sutiles. Adicionalmente, su estudio estaba basado en la lectura por parte de cinco médicos de urgencias, mientras que en nuestro estudio esta fue realizada únicamente por neurorradiólogos.

El estudio de Mclaughlin *et al.*^10^, utilizó el mismo tipo de computador tableta que en nuestro estudio. Evaluaron la concordancia interobservadores en el ASPECTS para observadores individuales, agrupando los puntajes ASPECTS en cuatro categorías (1-3, 4-7, 8-9 y 10). Se notaron concordancias interobservadores sustanciales para los neurorradiólogos y concordancias regulares para los radiólogos junior. Utilizaron dos neurorradiólogos en consenso para evaluar la concordancia utilizando el computador tableta, pero no calcularon las concordancias para neurorradiólogos individuales ni con computador tableta ni con la imagen de referencia. Adicionalmente, no calcularon las concordancias intraobservadores. Para compararlo con este estudio, además del ASPECTS dicotomizado, calculamos las concordancias en los puntajes ASPECTS categorizados con las mismas categorías para el grupo general de radiólogos, el cual se presenta en la Tabla 1. Los valores para los radiólogos individuales no se presentan porque pocos pacientes por radiólogo en cada categoría tenían valores kappa no confiables (*todos p* >0.05). En nuestro estudio, todas las categorías intraobservadores fueron clasificadas como buenas o moderadas, con valores kappa de 0.43 a 0.68. Al igual que en el estudio de McLaughlin, se observaron valores kappa más altos para las categorías 1-3 que 10 y fueron más bajos para las categorías 4-7 y 8-9. 

## Conclusión

No se encontró superioridad de un sistema de lectura por encima del otro en las concordancias de las variables clínicas evaluadas, al igual que en nuestra evaluación anterior, en la que todas las variables relacionadas eran estadísticamente equivalentes (al umbral seleccionado de 10%), al utilizar los sistemas de lectura Médico-IMPAX y Tableta-XERO. Este es un hecho relevante que presenta evidencia de que los sistemas de lectura Médico-IMPAX y Tableta-XERO podrían ser intercambiables, sin perder confiabilidad, cuando los neurorradiólogos utilizan soluciones móviles para interpretar imágenes de TAC de cráneo simple de pacientes con síntomas de ACV agudo. Por lo tanto, esto aumenta la eficiencia, suministro y disponibilidad de servicios de teleACV para poblaciones desatendidas.

En el diseño estadístico de este estudio, el radiólogo y los sistemas de lectura eran factores fijos, ya que no fueron seleccionados aleatoriamente; por lo tanto, nuestros resultados solo aplican a ellos. No obstante, dado que los neurorradiólogos son radiólogos muy especializados, esperamos que nuestros resultados puedan ser generalizados a otros neurorradiólogos. De igual manera, los sistemas de lectura en radiología deben cumplir con DICOM, lo cual nos permite generalizar nuestros resultados a otro software de lectura u otros medios médicos de visualización. En contraste, la pantalla del computador tableta podría ser muy diferente, de manera que nuestros resultados aplican únicamente a los que utilizan pantallas “retina”.

En nuestro estudio, la lectura de la TAC de cráneo simple con un computador Tableta se realizó a través de una conexión Wi-Fi; para un enfoque más realista en nuestro país, en el que el Wi-Fi público no siempre está disponible, es necesaria una evaluación del costo y tiempo para la transferencia de la imagen por las redes de telefonía móvil, tales como una red 4G.
